# Validity and Reliability of the Korean Version of the Fear of COVID-19 Scale

**DOI:** 10.3390/ijerph18147402

**Published:** 2021-07-11

**Authors:** Jeong-Won Han, Junhee Park, Hanna Lee

**Affiliations:** 1 College of Nursing Science, Kyung Hee University, Seoul 02447, Korea; hjw0721@khu.ac.kr; 2 Department of Nurinsg, Dongnam Health University, Suwon-si 16328, Korea; junheepark@dongnam.ac.kr; 3 Department of Nursing, Gangneung-Wonju National University, Gangneung-si 26403, Korea

**Keywords:** COVID-19, infections, Republic of Korea, fear

## Abstract

Background: The present study aimed to translate the Fear of COVID-19 Scale (FCV-19S) into the Korean language and test the validity and reliability of the translated Korean version. Methods: An online questionnaire survey was conducted with 300 adults (aged ≥19 years) living in South Korea. The data collection period was 1 June to 15 October 2020. The Korean version of the FCV-19S (KFCV-19S) was tested in terms of content validity, construct validity, criterion validity, item response theory, and reliability. Results: When the content and construct validity of the FCV-19S was tested, the results showed that all items could be retained in the Korean version. When the criterion validity was tested based on correlation analysis between the KFCV-19S and the State-Trait Anxiety Inventory, the results showed a positive correlation (r = 0.53, *p* < 0.001). Item suitability test results showed that all items were within the reference value of 0.5–1.5. Internal consistency reliability test results showed a Cronbach’s alpha of 0.81. Conclusion: The applicability of the KFCV-19S for identifying the level of fear Korean people experience regarding COVID-19 was verified. This tool is recommended for use in future assessments of Korean populations regarding levels of fear and anxiety regarding COVID-19.

## 1. Introduction

The coronavirus disease 2019 (COVID-19) pandemic has become one of the most significant health crises of the present generation [1]. Globally, the number of confirmed COVID-19 cases, which continues to increase rapidly, reached 120,176,364, and the number of COVID-19-related deaths reached 2,659,578, as of 16 March 2021 [2]. The World Health Organization (WHO) reported that, in the 2002–2003 outbreak of severe acute respiratory syndrome (SARS), the total number of confirmed cases was 8096 and the fatality rate was 9.6%, while the total number of confirmed Middle East respiratory syndrome (MERS) cases between 2012 and 2019 was 2494 and the fatality rate was 34.4% [3]. On the other hand, the number of confirmed COVID-19 cases worldwide ranges in the millions, and its fatality rate and spread may be higher and faster, respectively, when compared to recent infectious disease outbreaks [4]. COVID-19 is caused by a virus that can infect humans and various animals [3]. The virus’ form is very favorable for binding to cell membranes through activation of the viral spike proteins; additionally, it is continually mutating and has high transmissivity. Consequently, people are experiencing high levels of fear regarding COVID-19 [5].

The COVID-19 pandemic has affected people of all nations, continents, races, and socioeconomic groups. The responses that have been implemented, such as the quarantining of entire communities, the closing of schools, social isolation, and the implementation of shelter-in-place orders, have abruptly changed daily life [6]. In particular, the prolongation of the COVID-19 outbreak is causing many people to experience problems in regard to performing activities of daily living, as they are developing symptoms such as psychological tension and anxiety while attempting to manage the stress caused by the changes the anti-COVID-19 measures have made to their daily lives [7]. Further, people are exhibiting distrust and anger toward others as a result of anxiety regarding COVID-19, and confirmed patients and patients placed under isolation are experiencing psychological fear regarding the prognosis of the disease [7]. Moreover, medical professionals are reporting a fear of contracting COVID-19 through contact with infected patients and as a result of inadequate access to protective equipment [1]. Unfortunately, fear may amplify the damage of the disease itself [8,9]; owing to the nature of the COVID-19 pandemic, such fears are currently prevalent worldwide and, in some cases, have fostered stigma [10]. Fear of disease is directly associated with the transmission rate and medium of the disease in question (in the case of COVID-19, rapid and invisible, respectively), as well as the associated morbidity and mortality rates [11]. Such fear fosters psychosocial issues such as stigmatization, discrimination, and a sense of loss [12]. During the 2003 SARS outbreak, people in Hong Kong coined the term “SARS phobia” to describe the threat and fear of SARS infection [13]; meanwhile, the term “MERS phobia” was used in South Korea to express the unprecedented level of fear and terror the people were feeling regarding the MERS outbreak [14].

People have been found to experience psychological changes in response to disease outbreaks; these range from “being worried” in the early stages of the outbreak to “concern about one’s self and family becoming infected” [15]. Those with a relatively high level of fear regarding an infectious disease tend to be less-able to cope with the disease and manage their personal and social lives [15]. From this perspective, identifying people’s level of fear regarding COVID-19 and applying appropriate psychological interventions is important for helping people to adopt healthy lifestyles during the pandemic and to smoothly adapt to their new personal and social lives when the pandemic ends. In particular, considering that South Korea was one of the earliest countries to experience a COVID-19 outbreak and is still addressing the pandemic, it is necessary to identify the level of fear of COVID-19 among South Koreans. However, there are few suitable measurement tools for performing such examinations among Korean populations. Ahorsu et al. [11] recently developed the Fear of COVID-19 Scale (FCV-19S), which is a seven-item tool designed to measure fear of COVID-19 among adults; it has been verified as having high validity and reliability. The FCV-19S has the advantage of being developed to consider various diseases and subjects, and its items were preliminarily verified by an expert panel (comprising a psychologist, virologist, health psychologist, psychiatrist, general physician, and nurse; [11]). The items were then verified with respect to various aspects regarding fear of COVID-19 by a secondary expert panel (comprising a health education specialist, pulmonologist, social psychologist, and sociologist; [11]). Accordingly, the purpose of the present study was: (1) to translate the FCV-19S (along with its advantages) into the Korean language, and (2) to test the validity and reliability of the translated version in relation to its ability to obtain basic data regarding the level of fear of COVID-19 among Korean populations.

## 2. Materials and Methods

### 2.1. Study Design

This study comprised a methodological study that aimed to translate the FCV-19S into the Korean language and test the validity and reliability of the translated version.

### 2.2. Study Population

The study population comprised adults aged ≥19 years who lived in South Korea, had no difficulties communicating, and who were not receiving (at the time of this research) neuropsychiatric treatments for conditions such as anxiety and depression. Considering the environmental characteristics of South Korea, the nationality of the participants was restricted to South Korean only. When, in validity analysis, factor analysis is used as the main analytical method, a common recommendation is that there should be at least 100 participants or an item to sample ratio of 1:5 [16]. In the present study, we performed preliminary confirmation of the factor structure of the tool using exploratory factor analysis (EFA), and then performed confirmatory factor analysis (CFA) to confirm the EFA results. Consequently, different study populations were used for the EFA and CFA. As a sample size of at least 100 is recommended for EFA (as mentioned above) and a sample size of at least 200 is recommended for CFA, the total study population was set to 300. However, considering a drop-out rate of 10%, we administered the questionnaire survey to a total of 330 participants. Overall, 300 questionnaires were returned from the 330 questionnaires distributed, giving a return rate of 90.9% and a response rate of 100%.

### 2.3. Measurement

#### 2.3.1. Fear of COVID-19 Scale

The FCV-19S is a measurement tool developed by Ahorsu et al. [11] that contains a total of seven items: (1) “I am most afraid of coronavirus-19”; (2) “It makes me uncomfortable to think about coronavirus-19”; (3) “My hands become clammy when I think about coronavirus-19”; (4) “I am afraid of losing my life because of coronavirus-19”; (5) “When watching news and stories about coronavirus-19 on social media, I become nervous or anxious”; (6) “I cannot sleep because I’m worrying about getting coronavirus-19”; and (7) “My heart races or palpitates when I think about getting coronavirus-19.” Each item is scored using a five-point scale. The total score has a range of 5–35 points, with higher scores indicating more severe fear of COVID-19.

#### 2.3.2. State-Trait Anxiety Inventory

This study used Spielberger’s State-Trait Anxiety Inventory Korean YZ form (STAI-KYZ), which is a self-report state-trait anxiety inventory originally developed by Spielberger [17] and subsequently modified and supplemented by Hahn et al. [18] to be suitable for Korean populations. Among the state and trait anxiety items developed by Spielberger [17], the present study used 20 items relating to state anxiety. Each item is graded using a four-point scale, with higher scores indicating higher levels of anxiety. Hahn et al. [18] measured the reliability of the tool, reporting a Cronbach’s alpha value of 0.93.

### 2.4. Data Collection Procedure

The data collection period was 1 June to 15 October 2020. Considering that this collection period coincided with the COVID-19 prevention and control stage in South Korea, Google Forms was used to administer the survey to consenting participants. Survey was conducted by convenience sampling among companies, schools, and religious facilities. The Internet URL was sent to the department head of the relevant institution. Before accessing the online questionnaire survey, the participants were presented with an informed consent form (ICF). The ICF contained information regarding the purpose of the study, stated that the survey results would not be used for purposes other than those predefined in the information sheet, assured the participants that their responses were anonymous and would remain confidential, clarified that each participant could withdraw from the study at any time, and provided contact information for the researcher. Participants who completed and submitted the ICF clicked the “next” button to begin the survey.

### 2.5. Data Analysis

To develop the Korean version of the FCV-19S (KFCV-19S), permission to use the original FCV-19S was obtained from the developer. Based on the tool translation and application guidelines recommended by the WHO [19] for adapting tools developed in English to other languages and cultures, the items contained in the final tool developed in the present study were created through a process of preliminary translation, expert panel review, back translation, and pre-testing. The preliminary translation process, two researchers independently conducted the preliminary translation, and then, for each of the translated sentences, performed the process of consensus building to ensure that literal translation was avoided and that the intended meaning of each sentence was accurately conveyed. The translated draft was then back-translated by a professor of English language and literature, and a nursing expert whose native language was English compared the back-translated material to the original version to check the consistency of the meaning. Areas where the meanings differed were revised, and a pilot survey was conducted on 10 adults to test the accuracy of these revised areas. Subsequently, content validity was verified by two professors of nursing and one nursing PhD student. The final questionnaire, with verified content validity, was then tested for readability and comprehensibility by administering it to three adults from the pilot survey, and revisions were made as necessary. IBM Statistics AMOS 22.0 (IBM Corp, Armonk, NY, USA) and SPSS Statistics 26.0 (SPSS Inc., Chicago, IL, USA, 2019) were employed to analyze the collected data. The participants’ general characteristics were confirmed using frequencies, percentages, means, and standard deviations. In the content validity test, we used a content validity index of ≥0.8 as the selection criterion for items. After conducting random case sampling of the data for all participants, EFA was conducted on the data for 100 participants. To consider the correlation between the factors obtained through principal axis factor analysis, varimax rotation was applied. In EFA, a Kaiser-Meyer-Olkin (KMO) value of >0.50 is considered to indicate feasibility for conducting factor analysis; meanwhile, factor loading values are recommended to be at least 0.4, eigenvalues should be 1.0 or higher, and cumulative variance should be 50–60% or higher [20]. Meanwhile, for each variable, a communality below 0.3 indicates that the variable may have little in common with any of the other variables and should be excluded from the analysis [21]. Next, we conducted CFA using the data for the remaining 200 participants to test the measurement equivalence of the KFCV-19S. After reviewing variate normality, bootstrapping was employed for estimates that did not satisfy multivariate normality. The goodness-of-fit index (GFI) of the model was confirmed using normed χ^2^ (NC), standardized root mean residual (SRMR), Tucker–Lewis Index (TLI), and the Comparative Fit Index (CFI). To confirm the convergent validity of the factor construction, we applied the criterion of a standardized regression coefficient of ≥0.50 for each observed variable [22]. For criterion validity analysis, Pearson’s correlation coefficients for the KFCV-19S and the STAI-KYZ were calculated. For item response analysis, we used a polytomous item response model (Rasch model) to test the suitability of each item in the KFCV-19S and to determine whether the number of response categories was appropriate. For this analysis, jMETRIK 4.1.1 (jMetrik Item Analysis, Virginia, USA) was used, with the statistical significance level set to α = 0.05. Moreover, outfit and infit were examined to determine the degree to which the data deviated from the one-dimensional expected values of the Rasch model [23]. For outfit and infit, the reference value of 0.5–1.5 suggested by Linacre [24] was applied, and any item that deviated from this value was considered to be unfit. In addition, an item information curve was used to determine whether the items accurately measured the desired attribute level. The GFI of the rating scale was verified using the outfit and infit indices for the response categories [25]. The internal consistency was confirmed using Cronbach’s α coefficient; Cronbach’s α values of 0.70 and 0.80 are considered to indicate good internal consistency reliability, while values of 0.80 and 0.90 are considered to indicate very high consistency [26].

## 3. Results

### 3.1. Participants’ General Characteristics

The study population comprised 98 males (32.7%) and 202 females (67.3%). The number of participants aged <30, 30–39, 40–49, and ≥50 years was 80 (26.7%), 103 (34.3%), 52 (17.3%), and 65 (21.7%), respectively. Regarding highest education level obtained, the number of high school graduates, two-year college graduates, and four-year university graduates or higher was 57 (19.0%), 46 (15.3%), and 197 (65.7%), respectively. Regarding religion, the number of participants who reported that they were Christian, Buddhist, Catholic, and no religion was 54 (18.0%), 43 (14.3%), 69 (23.0%), and 134 (44.7%), respectively. Regarding marital status, 186 participants (62.0%) were married and 114 participants (38.0%) were single. Regarding area of residence, the number of participants residing in Seoul, Gyeonggi-do and Incheon, Gangwon-do, Gyeongsangnam-do, Gyeongsangbuk-do, Chungcheongbuk-do, and Ulsan was 64 (21.3%), 50 (16.7%), 4 (1.3%), 92 (30.7%), 39 (13.0%), 2 (0.7%), and 49 (16.3%), respectively. Regarding occupation, the number participants who were managerial workers, professionals, office workers, service workers, and others was 45 (15.0%), 130 (43.3%), 35 (11.7%), 28 (9.3%), and 62 (20.7%), respectively. For the question concerning whether they had been tested for COVID-19, 83 participants (29.7%) responded “yes” and 217 participants (72.3%) responded “no.” For the question of whether they knew anyone who had been confirmed as having COVID-19, 27 participants (9.0%) responded “yes” and 273 participants (91.0%) responded “no” (Table 1).

### 3.2. Validity Tests

#### 3.2.1. Content Validity

In the present study, content validity was verified by two professors of nursing and one nursing PhD student. Item suitability and sufficiency and the representativeness of the sub-domain items were tested using item-level content validity index (I-CVI) and scale-level content validity index (S-CVI), and S-CVI/Ave was consequently verified. For both I-CVI and S-CVI/Ave, the overall total for the seven items used in the present study was 1.00, respectively, indicating suitable content validity.

#### 3.2.2. Construct Validity

##### Exploratory Factor Analysis

EFA was performed on all seven items using 100 sets of data, for which principal component factor analysis and varimax rotation were applied. The KMO value for the suitability of the factor analysis samples was 0.85, which was higher than the reference value of 0.80, while Bartlett’s test for sphericity showed an approximate χ^2^ value of 1003.37 (df = 21, *p* < 0.001), indicating that use of factor analysis was suitable. Factor analysis of all seven items found one factor with an initial eigenvalue of ≥1.0. All items showed a communality value of ≥0.50 and satisfied the factor loading criterion of ≥0.40. After the EFA, all seven initial items were retained (Table 2).

##### Confirmatory Factor Analysis

**Figure 1:**
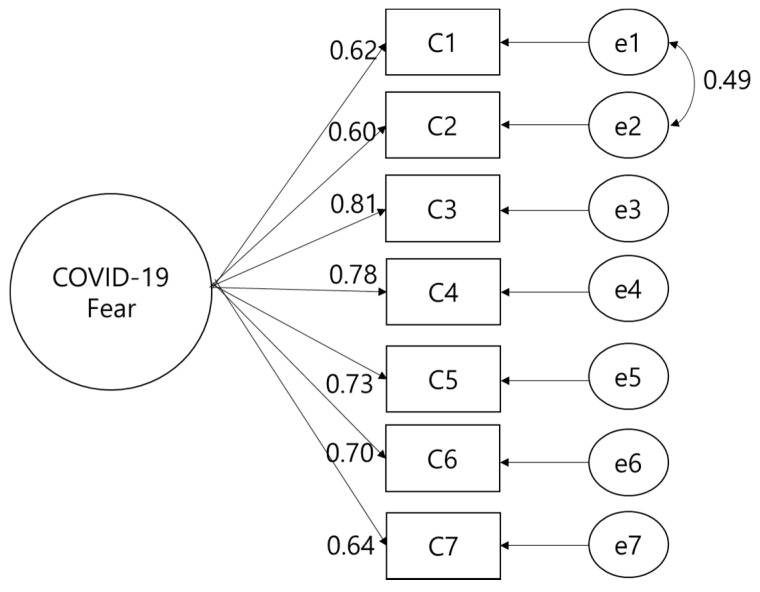
Confirmatory factor analysis.

CFA was performed on seven items on one subfactor verified by the preliminary content validity test. Assessment of the model’s GFI showed an NC of 3.04, an SRMR of 0.05, a TLI of 0.87, and a CFI of 0.92, indicating satisfactory GFI. The convergent validity and discriminant validity of the final model were confirmed. The standardized regression coefficient of all items was statistically significant (0.60~0.81), while the concept reliability was 0.98 and the average variance extracted index was 0.90 (Figure 1).

#### 3.2.3. Criterion Validity

Regarding analysis of criterion validity, correlation analysis between the KFCV-19S and the STAI-KYZ showed a positive correlation (r = 0.53, *p* < 0.001).

### 3.3. Item Response Theory Test

To apply the item response theory, it was necessary to verify the assumption that the scale was one-dimensional. The factor analyses results presented above showed that the KFCV-19S measures a single factor with respect to content, thus confirming the assumption that the scale was one dimensional. When the KFCV-19S was tested for item suitability, all items values were within the reference value of 0.5–1.5 (Table 3).

**Figure 2:**
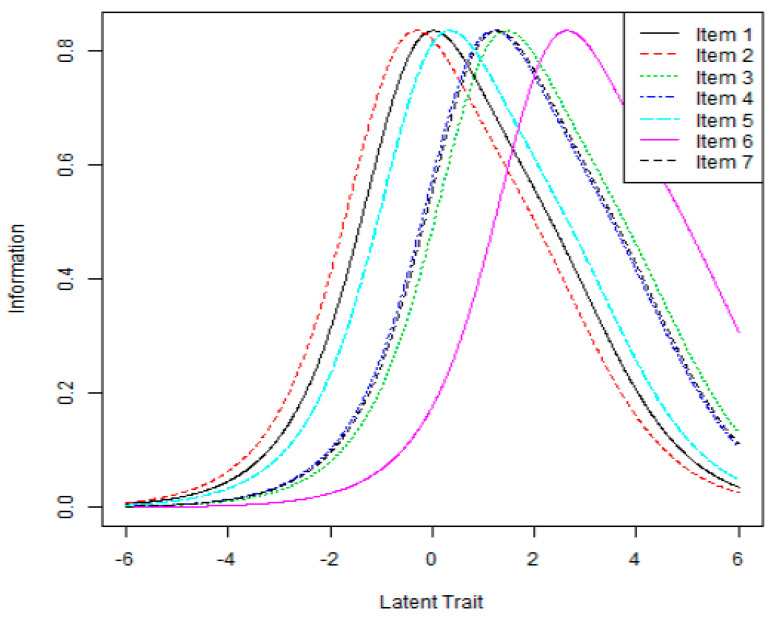
Item information curves.

The item information curve showed that the highest part of the curve appeared at a fear level of 0–3, which indicated that the items in the KFCV-19S are most suitable for subjects with a fear level between 0 and 3 (Figure 2). Rating scale analysis was performed to determine whether the five-point response categories of the KFCV-19S adequately discriminated the subjects’ responses. The results showed that each item of the KFCV-19S satisfied the infit and outfit criteria for response category (Table 4); therefore, it was determined that the five-point response categories of the KFCV-19S adequately discriminate the subjects’ responses.

### 3.4. Reliability Analysis

When the internal consistency reliability of the KFCV-19S was tested, the subfactor showed a Cronbach’s alpha of 0.81 (Table 1).

## 4. Discussion

First, content validity test results confirmed that the items in the KFCV-19S are equivalent to the seven items that were originally developed for the FCV-19S by Ahorsu et al. [11]. This indicates that there is little inter-culture difference regarding individuals’ perceptions of their fear of COVID-19. Moreover, as in Ahorsu et al. [11], both the EFA and CFA results for the KFCV-19S satisfied the statistical reference values, indicating that there should be no issues confirming the validity of this measurement tool. 

Second, correlation analysis involving the KFCV-19S and the STAI-KYZ [18], which was performed to analyze criterion validity, returned a positive correlation. Criterion validity tests determine the tested scale’s relation to a gold standard tool that features the same concept as the target scale [27]; however, since the only tool that can be used to determine the criterion validity of the KFCV-19S is the original FCV-19S developed by Ahorsu et al. [11], correlation analysis was performed using the STAI-KYZ [18], which measures state anxiety regarding infectious diseases. Notably, Ahorsu et al. [11] also experienced limitations regarding using a gold standard tool and, as a result, they used the Hospital Anxiety and Depression Scale [28] for their criterion validity test. Moreover, applying common criterion validity assessment criteria (that a correlation coefficient of 0.60–0.80 indicates “high validity” and one of 0.80–1.0 indicates “very high validity”; [29]) shows that the KFCV-19S can be considered a valid tool for measuring level of fear regarding COVID-19 among Korean populations. Overall, the above study results indicate that the KFCV-19S is a measurement tool with proven validity.

Third, for the item analysis in the present study, the Rasch model used for two-item scales was expanded to suit a multi-item scale, and was used to analyze the seven items of the KFCV-19S based on difficulty, discrimination, suitability, and item information curve. Difficulty is a concept associated with the level of fear of COVID-19; items with low difficulty could yield high scores even if the level of fear of COVID-19 is not severe, whereas items with high difficulty could yield high scores when the level of fear of COVID-19 is severe. When the difficulty of each item in the KFCV-19S was analyzed, the results ranged from −1.50 to 1.94, indicating an even distribution from low to high difficulty. Accordingly, it was determined that the KFCV-19S features a sufficient measurement range to account for the various levels of fear of COVID-19, from low to severe. Moreover, item suitability was tested as an indicator of the presence of specific patterns in responses or the appearance of unexpected values, such as outliers. The results for all seven items were within the acceptable range for rating scales (0.5–1.5) and, thus, item suitability was determined to be satisfies the suitability criteria [23].

Lastly, regarding the internal consistency reliability of the KFCV-19S, the Cronbach’s α was 0.81, which confirmed the homogeneity criteria of the test.

This study aimed to develop the KFCV-19S, a tool for identifying the level of fear regarding infectious disease among people in South Korea. Such a tool is needed as the COVID-19 outbreak in the country continues. However, 65.7% of the subjects of this study were found to have graduated from a four-year university, which may limit the validity of the questionnaire used for people with low education levels. Therefore, in future studies, it may be necessary to expand the study to subjects with various educational levels.

## 5. Conclusions

This study translated the FCV-19S, developed by Ahorsu et al. [11], into the Korean language and tested the validity and reliability of the translated Korean version in regard to its ability to obtain basic data concerning the fear and anxiety Korean adults feel regarding COVID-19. The study results confirmed that all items in the KFCV-19S have acceptable reliability and validity, thereby confirming the applicability of the tool for measuring fear of COVID-19 among Koreans. The significance of the present study is that it tested the validity and reliability of the KFCV-19S among Koreans, and this tool is expected to be actively used in the future for assessing the level of fear patients have regarding COVID-19. Studies on the development of education and programs for reducing fear caused by COVID-19, and assessments of the effectiveness of such education and programs, are recommended for future research.

## Figures and Tables

**Table 1:** Participants’ general characteristics.

Variables	Categories	N (%)
Gender	Male	98 (32.7)
	Female	202 (67.3)
Age (years)	<30	80 (26.7)
	30–39	103 (34.3)
	40–49	52 (17.3)
	>50	65 (21.7)
Level of education	Under high school	57 (19.0)
	College (2 year)	46 (15.3)
	University (4 year)	197 (65.7)
Religion	Christianity	54 (18.0)
	Buddhism	43 (14.3)
	Catholicism	69 (23.0)
	None	134 (44.7)
Marital status	Married	186 (62.0)
	Not married	114 (38.0)
Residence	Seoul	64 (21.30)
	Gyeonggi-do and Incheon	50 (16.7)
	Gangwon-do	4 (1.3)
	Gyoensangnam-do	92 (30.7)
	Gyeongsangbuk-do	39 (13.0)
	Chungcheongbuk-do	2 (0.7)
	Ulsan	49 (16.3)
Job	Managerial workers	45 (15.0)
	professionals	130 (43.3)
	office workers	35 (11.7)
	service workers	28 (9.3)
	others	62 (20.7)
COVID 19 screening test	Yes	83 (29.7)
	None	217 (72.3)
Confirmed cases of COVID 19	Yes	27 (9.0)
	None	273 (91.0)

**Table 2:** Exploratory factor analysis of fear of COVID-19 Scale.

Items	Factor Loading	Mean ± SD
I am most afraid of coronavirus-19.	0.80	3.10 ± 1.17
It makes me uncomfortable to think about coronavirus-19.	0.73	3.30 ± 1.18
My hands become clammy when I think about coronavirus-19.	0.83	2.14 ± 1.13
I am afraid of losing my life because of coronavirus-19.	0.81	2.30 ± 1.15
When watching news and stories about coronavirus-19 on social media, I become nervous or anxious	0.80	2.89 ± 1.11
I cannot sleep because I’m worrying about getting coronavirus-19.	0.73	1.56 ± 0.91
My heart races or palpitates when I think about getting coronavirus-19.	0.70	2.25 ± 1.18
Eigenvalue	4.01	
% of variance	57.30	
% of cumulative	57.30	
Cronbach’s alpha	0.81	

Note: SD = Standard deviation.

**Table 3:** Item suitability index.

Item	Difficulty	Infit	Outfit
1	−1.05	1.08	1.14
2	−1.50	1.07	1.03
3	0.63	0.76	0.68
4	0.37	0.88	0.89
5	−0.70	0.91	0.90
6	1.94	0.97	0.81
7	0.32	1.32	1.49

**Table 4:** Item suitability index.

Item	Category	Infit	Outfit
1	0	-	-
	1	1.13	1.20
	2	1.07	1.03
	3	1.08	1.31
	4	1.25	1.19
2	0	-	-
	1	0.74	0.73
	2	1.02	0.96
	3	1.24	1.03
	4	1.44	1.47
3	0	-	-
	1	1.03	0.68
	2	0.79	0.67
	3	0.80	0.52
	4	0.62	0.65
4	0	-	-
	1	1.05	1.27
	2	0.78	0.65
	3	0.60	0.59
	4	0.82	0.80
5	0	-	-
	1	0.76	0.72
	2	0.95	0.93
	3	1.04	1.00
	4	1.26	1.22
6	0	-	-
	1	1.09	0.56
	2	0.77	0.58
	3	0.72	1.27
	4	0.51	0.50
7	0	-	-
	1	1.18	1.16
	2	1.17	1.41
	3	1.32	1.46
	4	0.63	0.71

## Data Availability

No new data were created or analyzed in this study. Data sharing is not applicable to this article.
